# The evolving role of nonprofit hospitals and health systems in improving community health

**DOI:** 10.3389/fpubh.2026.1745949

**Published:** 2026-02-09

**Authors:** Simone R. Singh, Michael Shepherd

**Affiliations:** Department of Health Management and Policy, School of Public Health, University of Michigan, Ann Arbor, MI, United States

**Keywords:** anchor institution, community benefit, community health, community partnerships, hospitals

## Abstract

As the healthcare landscape evolves, hospitals and health systems in the United States (U.S.) are increasingly focusing on factors beyond traditional clinical care, recognizing that social determinants such as housing, nutrition, transportation, and education profoundly influence health outcomes. Historically, hospitals primarily provided medical services within their facilities, but their role has expanded to include efforts that address both patients’ social needs and the overall well-being of the communities they serve. For nonprofit hospitals in the U.S., this shift is driven not only by the mission of these hospitals but also by legal requirements tied to their tax-exempt status; they must demonstrate community benefit through financial investments reported on their annual tax return, the Internal Revenue Service (IRS) Form 990 Schedule H. However, the community benefit reporting categories included on Schedule H are limited and do not fully capture the breadth of hospital contributions to community health. Nonprofit hospitals deliver essential, often unprofitable services, provide uncompensated care to uninsured patients, address the social needs of their patients, participate in cross-sector collaborations, contribute to healthcare workforce development, and serve as anchor institutions within their communities. By leveraging their resources and influence, many nonprofit hospitals in the U.S. are transforming from traditional healthcare providers into community leaders, actively promoting health equity and improving population health on a larger scale. This expanded role reflects a holistic approach to health that acknowledges the complex interplay between medical care and social factors.

## Introduction

As the healthcare landscape in the United States (U.S.) continues to shift, hospitals and health systems are increasingly being called upon to look beyond traditional clinical care and address broader determinants of health that impact the health and well-being of the populations they serve. Traditionally, the primary role of hospitals in the U.S. has been to deliver high-quality clinical services to individual patients who sought care within their facilities (1). In recent decades, however, the role of hospitals has evolved significantly. Increasingly, these organizations recognize that factors outside their four walls, such as housing, nutrition, transportation, and education, play a major role in improving the health of individual patients and patient populations (2). As a result, hospitals in the U.S. have become more engaged in initiatives aimed at improving both patients’ health-related social needs and the overall health and well-being of the communities they serve (3).

For nonprofit hospitals, which represent more than half of all hospitals in the U.S., community engagement is not only a core part of their mission, but also a legal obligation. In the U.S., these organizations typically receive substantial tax exemptions, and in return, they are required to demonstrate contributions to the well-being of their communities in the form of community benefit (4). Community benefit refers to the activities and programs that hospitals provide to improve health in their communities, beyond basic patient care. These are initiatives that aim to address the health needs of the broader public, especially vulnerable populations.

In the U.S., nonprofit hospitals are required to report their community benefit activities on their annual tax return for nonprofit organizations, Internal Revenue Service (IRS) Form 990 Schedule H. Schedule H offers a standardized method for reporting investments by hospitals in community health (5). In its current form, Schedule H categorizes hospital community benefit into two primary areas: (1) financial assistance and means-tested government programs, and (2) other community benefits. The first category encompasses charity care (i.e., care provided to patients who do not have insurance) and the unreimbursed costs of hospital participation in government health programs such as Medicaid and the Children’s Health Insurance Program (CHIP). The second category includes a broader range of activities, such as community health improvement services, community benefit operations, health professions education, and medical research—all of which extend beyond direct clinical care. While Schedule H provides important transparency into hospital activities aimed at improving the health of patients and communities, the reporting categories are limited in scope (6, 7). In reality, we argue that the positive impact of nonprofit hospitals on community health and well-being is far broader than what can be captured on this form alone, encompassing initiatives and outcomes that may not be reflected in Schedule H documentation.

This manuscript examines the evolving role of nonprofit hospitals and health systems in advancing community health—not only as providers of medical care, but as integral members of the broader health ecosystem. It begins by evaluating hospitals’ commitment to offering essential, often financially unprofitable services, providing of uncompensated care, and addressing health-related social needs through screening and direct support initiatives. The discussion then expands to consider hospitals’ broader contributions to community health, such as their involvement in cross-sector collaborations aimed at improving health, commitment to healthcare workforce development, and their function as anchor institutions within communities. Ultimately, this perspective illustrates how nonprofit hospitals are transforming from traditional healthcare providers into pivotal community partners and leaders.

## Hospitals and health systems as providers of medical care

In the U.S., hospitals and health systems contribute to the health of their communities through the provision of clinical services that are relatively unprofitable and therefore frequently lose the organization money. By providing these services despite the lack of financial incentives, hospitals are directly contributing to improving the health of individual patients. Examples of relatively unprofitable services in the U.S. include emergency services (e.g., trauma centers), birthing rooms, psychiatric services, substance use disorder services, transplant services, and services provided at burn centers (8–10). Prior research has shown that nonprofit hospitals are more likely than their for-profit counterparts to offer services that are relatively unprofitable. At the same, nonprofit hospitals are less likely to adopt or discontinue services based on changes in service profitability, which can impact service availability in a community over time (9, 11). Unlike for-profit hospitals, which aim to generate profits for their owners and therefore limit the provision of money-losing services to the extent possible, nonprofit hospitals have incentives to provide relatively unprofitable services as part of their mission as well as their legal obligation to provide community benefit in the form of free and reduced cost care.

In 2010, major health reform passed in the U.S. The Affordable Care Act (ACA) contains numerous provisions, including substantial expansions of health insurance coverage to U.S. residents who were previously uninsured. Since the passage of the ACA, despite the expansion of health insurance coverage, general patterns of hospitals’ provision of relatively unprofitable services have changed relatively little (11). However, increased financial pressures in the form of stagnant reimbursement rates and increasing costs of care may reduce hospitals’ ability to provide such services going forward. Research has shown that strong financial performance allows hospitals to continue providing relatively unprofitable services while hospitals in weaker financial condition have been found to reduce their provision of such services (12). Importantly, however, hospitals do not necessarily cut all unprofitable services equally; they often reduce investment in certain areas while increasing resources in others, often guided by community health needs assessments or changing local priorities.

Hospitals and health systems also contribute to the health of their communities through the provision of uncompensated care, that is, healthcare services provided by the hospital without receiving payment from the patient or a third-party payer. While most U.S. hospitals, including for-profit hospitals, provide some level of uncompensated care, uncompensated care is a central expectation for nonprofit hospitals, which must provide such care as part of their community benefit spending to maintain their tax-exempt status. On average, nonprofit hospitals dedicate more than 8 % of their total expenditures to community benefit activities, with a significant amount of spending allocated to uncompensated care (13–16).

Provision of uncompensated care was especially critical prior to the passage of the ACA due to high rates of uninsurance in many U.S. communities. While the ACA has significantly reduced the number of uninsured individuals, hospitals’ overall community benefit spending has remained relatively stable. Hospitals in states that expanded Medicaid under the ACA, saw a decline in uncompensated care, largely due to a decrease in the number of uninsured patients. However, the resulting savings in financial assistance costs were often at least partially offset by increased unreimbursed costs from providing care to patients covered by Medicaid (15, 17). Hospitals serving large proportions of Medicaid patients face ongoing challenges, especially in light of anticipated future federal policy changes. The One Big Beautiful Bill (OBBB) Act of 2025 is projected to reduce U.S. healthcare funding by over $1 trillion, with most of these cuts targeted at Medicaid. Such funding reductions could threaten hospitals’ ability to sustain current levels of uncompensated care and other community benefit initiatives.

Finally, hospitals and health systems enhance community health by actively screening patients for health-related social needs (HRSNs) and providing supportive services. HRSNs encompass factors such as food insecurity, housing instability, and transportation barriers, all of which can profoundly affect patient care and health outcomes (18). When patients are identified through screening as experiencing one or more HRSNs, hospitals respond by offering assistance through in-house support services or by connecting them with relevant community resources (19, 20). Developing internal programs, such as food pantries, transportation assistance, or financial counseling, may be limited by the amounts and levels of available funding, staffing, or expertise (21, 22). To overcome these limitations, many hospitals establish partnerships with community-based organizations that specialize in areas like housing, food security, legal aid, and employment support. These collaborations allow hospitals to refer patients to agencies equipped to provide targeted assistance, ensuring that patients’ social needs are addressed in a coordinated and effective manner.

Since the passage of the ACA, HRSN screening has become increasingly commonplace in hospitals and health systems including both nonprofit and for-profit organizations (19, 20). The ACA introduced incentives for hospitals to reduce preventable readmissions and improve community health, leading to more widespread and systematic adoption of HRSN screening practices (23). In addition, payers have begun to require such screenings, further accelerating implementation across healthcare settings.

## Hospitals and health systems as members of the health ecosystem

Hospitals and health systems in the U.S. also contribute to the health of their communities through community health investments and partnerships (3). Partnerships between hospitals, public health organizations, and community stakeholders are crucial in identifying key community health needs and developing innovative strategies that promote wellness, prevention, and equitable access to care for all populations (24).

Since the passage of the ACA, nonprofit hospitals in the U.S. are required to conduct regular Community Health Needs Assessments (CHNAs) as part of their obligations for maintaining tax-exempt status (25). A CHNA is a systematic process used to identify and evaluate the health issues and needs of a specific population or community. The goal is to gather data and insights that can guide policies, programs, and resource allocation to improve overall health outcomes in that community. While the minimum requirement is to seek input from local public health agencies during the CHNA process, in practice many hospitals go further by collaborating closely with local public health officials and a range of community partners (26–29). This collaborative approach not only strengthens the assessment process but also helps ensure that the identified health priorities reflect the needs and perspectives of the broader community (30).

Since the passage of the ACA, nonprofit hospitals are also required to develop an implementation strategy as part of the CHNA process that addresses the needs identified in the assessment (25). This strategy outlines specific goals, initiatives, and actions the hospital will undertake to respond to the priority health issues revealed through the CHNA. Hospitals often collaborate with public health agencies, community partners, and other local stakeholders to design effective programs, allocate resources, and establish metrics to track progress over time (26). Hospitals may sponsor screenings, vaccination drives, educational campaigns, and initiatives to address chronic disease prevention and management.

Other examples of how hospitals engage in community health partnerships are through providing leadership and financial support, including grants and direct funding. Often acting as conveners, hospitals bring together diverse community organizations to foster collaboration and address shared health priorities. Hospitals also play a role in advocating for local, state, or national policies that support population health. Through these hospital-community partnerships, hospitals help build local capacity, implement health programs, and advance community-wide health goals.

Hospitals and health systems also contribute to the health of their communities through investing in healthcare workforce education, retention, and recruitment. Many hospitals have invested in workforce development programs to counteract workforce shortages and help train the next generation of healthcare workers. Such efforts have become increasingly important during and since the COVID-19 pandemic, when many healthcare professionals experienced heightened burnout and often sought out other employment opportunities (31, 32) as well as for rural hospitals, which persistently struggle with both recruitment and retention (33–35).

Broadly, workforce programming by nonprofit hospitals is focused on either education or retention among current employees, both medical and administrative, or the recruitment of new employees. For education and retention, successful workforce programming includes opportunities for on-site and in-classroom training as well as advance degree attainment paid for by the hospital, novel financial incentives (e.g., housing or relocation stipends) and work-life balance programming (e.g., flexible working hours and remote work), as well as opportunities for current employees to advance their careers within the hospital organization in new roles (36, 37). These efforts foster a sense of investment in and long-term support of employees, motivating them to invest in their own skills in ways that promote patient health and produce workforce stability for the hospital.

Recruitment efforts also take on a variety of forms from informal social media-based messaging to more formal partnerships (38–40). Some hospitals have established pipeline programs with students at local high schools, community colleges, and universities (41–43). Others have gone as far as to establish their own nursing and medical training programs as tools of recruitment, either truly on their own or via academic and community partnerships (40, 44, 45). Further, many hospitals use academic medical training and residency programs to recruit new talent into the hospital, a practice that is especially valuable for rural providers around the world (46–48).

Finally, nonprofit hospitals and health systems often contribute to the health of their communities by serving as anchor institutions. Anchor institutions are large, place-based organizations that invest in their communities, including the health of their communities, as a way of doing business (49, 50). Hospitals and health systems, sometimes termed anchor “meds,” commit substantial financial, human, and intellectual resources to support their communities, address social challenges, and improve community well-being and development (51, 52). As anchor institutions, nonprofit hospitals function not only as providers of healthcare, but also as economic engines that contribute to community stability, resilience, and long-term prosperity.

Nonprofit hospitals and health systems often rank among the largest employers in their communities, providing stable jobs, income, and, in the U.S., health insurance for thousands of residents across a wide range of roles—from clinical and administrative staff to support and technical services (51). Beyond employment, hospitals fuel the local economy by purchasing goods and services from nearby businesses, such as food suppliers, maintenance contractors, and medical equipment providers (52). Directing institutional purchasing towards local businesses helps stimulate small business growth and keeps economic activity within the community. Finally, hospitals frequently support the development of community infrastructure by providing resources and expertise to build local community capacity through place-based investments (53).

## Discussion

Many nonprofit hospitals and health systems care deeply about their communities and are invested in improving community health in numerous ways. While uncompensated care provision often garners the most attention, especially amid ongoing debates in the U.S. about whether nonprofit hospitals deliver sufficient community benefit to justify their tax-exempt status (54, 55), the contributions of these institutions encompass much more. The focus on uncompensated care tends to eclipse the broad array of activities and investments that hospitals undertake to promote health and well-being within their communities. In reality, the impact of hospitals extends far beyond what is captured on IRS Form 990 Schedule H (56).

Nonprofit hospitals and health systems serve as anchor institutions within their communities, employers, conveners, and trusted health advocates. Their work in improving health outcomes often goes beyond direct patient care. Hospitals participate in and frequently lead collaborative efforts to address the social determinants of health, such as housing, education, food security, transportation, and economic stability. They partner with local schools, governments, public health agencies, and community organizations to launch initiatives that address upstream factors impacting health status. Examples include sponsoring mobile health clinics, supporting violence prevention programs, investing in affordable housing, launching workforce development initiatives, and advocating for healthy built environments. Many hospitals also play a critical role in disaster preparedness, response, and broader public health infrastructure, especially during crises such as the COVID-19 pandemic.

Given this multifaceted role, it becomes evident that the current reporting framework — IRS Form 990 Schedule H — provides only a partial glimpse of the true breadth and depth of nonprofit hospitals’ contributions to their communities. While Schedule H requires documentation of certain expenditures, it does not fully account for less tangible or indirect activities that are equally important for improving population health, nor does it consistently capture the work hospitals do through partnerships and coalitions or the outcomes achieved from those efforts. As a result, policymakers, regulators, and the public currently do not have access to a complete picture of how nonprofit hospitals deliver value to their communities, potentially undermining informed discussion about hospital accountability and the societal return on tax-exempt status.

To address these gaps, more comprehensive documentation and reporting standards are needed. Enhanced reporting would enable hospitals to more fully showcase the scope of their community health investments and interventions, including non-clinical and collaborative activities. Standardized, comprehensive community benefit reporting—potentially at the regional, state, or national levels—would allow for more accurate comparisons of hospital performance, facilitate the sharing of best practices, and provide a robust evidence base for evaluating policy initiatives. While many hospitals already produce detailed reports and narratives for local and regional stakeholders, these efforts are not systematically aggregated or benchmarked (56). By creating a more cohesive and transparent reporting framework, the sector could strengthen public trust, support continuous improvement, and ensure policymaker and community stakeholders have the information needed to assess and support hospitals’ vital role in advancing community health. Expanded documentation may be accomplished by requiring hospitals to enhance the content of the implementation strategies they compile as part of the CHNA. Hospitals could, for example, broaden the scope of initiatives featured by highlighting, in the form of explicit line-items or concrete metrics, the hospital’s nonclinical programming and partnerships, improve data collection and reporting to collect comprehensive health outcomes metrics, and make results more accessible through infographics, videos, and interactive dashboards.

## Data Availability

The original contributions presented in the study are included in the article/supplementary material, further inquiries can be directed to the corresponding author.
